# Population structure in Argentina

**DOI:** 10.1371/journal.pone.0196325

**Published:** 2018-05-01

**Authors:** Marina Muzzio, Josefina M. B. Motti, Paula B. Paz Sepulveda, Muh-ching Yee, Thomas Cooke, María R. Santos, Virginia Ramallo, Emma L. Alfaro, Jose E. Dipierri, Graciela Bailliet, Claudio M. Bravi, Carlos D. Bustamante, Eimear E. Kenny

**Affiliations:** 1 Instituto Multidisciplinario de Biología Celular (IMBICE) CCT-La Plata CONICET-CICPBA, La Plata, Buenos Aires, Argentina; 2 Facultad de Ciencias Naturales y Museo, Universidad Nacional de La Plata, La Plata, Buenos Aires, Argentina; 3 Universidad Nacional del Centro de la Provincia de Buenos Aires, FACSO, NEIPHPA, Quequén, Buenos Aires, Argentina; 4 Stanford University, Stanford, California, United States of America; 5 CENPAT CONICET, Puerto Madryn, Chubut, Argentina; 6 INECOA (Instituto de Ecorregiones Andinas) UNJu-CONICET, Instituto de Biología de la Altura, Universidad Nacional de Jujuy, San Salvador de Jujuy, Jujuy, Argentina; 7 Charles Bronfman Institute for Personalized Medicine, Icahn School of Medicine at Mount Sinai, New York, New York, United States; Recinto Universitario de Mayaguez Universidad de Puerto Rico, UNITED STATES

## Abstract

We analyzed 391 samples from 12 Argentinian populations from the Center-West, East and North-West regions with the Illumina Human Exome Beadchip v1.0 (HumanExome-12v1-A). We did Principal Components analysis to infer patterns of populational divergence and migrations. We identified proportions and patterns of European, African and Native American ancestry and found a correlation between distance to Buenos Aires and proportion of Native American ancestry, where the highest proportion corresponds to the Northernmost populations, which is also the furthest from the Argentinian capital. Most of the European sources are from a South European origin, matching historical records, and we see two different Native American components, one that spreads all over Argentina and another specifically Andean. The highest percentages of African ancestry were in the Center West of Argentina, where the old trade routes took the slaves from Buenos Aires to Chile and Peru. Subcontinentaly, sources of this African component are represented by both West Africa and groups influenced by the Bantu expansion, the second slightly higher than the first, unlike North America and the Caribbean, where the main source is West Africa. This is reasonable, considering that a large proportion of the ships arriving at the Southern Hemisphere came from Mozambique, Loango and Angola.

## Introduction

One of the most important applications of admixture research is to reduce bias of association studies, since it has long been known that the underlying genetic structure can produce a high percentage of false positive results due to differences in the genetic composition of cases and controls [1–6]. This bias occurs when the frequency of the case disease varies between populations, so that the probability of selecting affected individuals from specific subpopulations grows; thus, any allele with a higher frequency in the over represented population will show an association with the phenotype [7–9].

DNA studies allow researchers to analyze population differences as well as the ancestry of a given individual, whether following a single line of ancestry (uniparental markers) or focusing on the autosomal regions and the X chromosome, which reveal different ancestral components. There is great interest in identifying admixture to show continental ancestry in genetically divergent populations [10–14]. These methods have been applied to African-American populations [15–20](Zhu et al. 2005, Smith et al. 2004, Tian et al. 2008, Reich et al. 2005, Deo et al. 2007, Reich et al. 2007), and have been powerful in identifying ancestry in Latin American populations [21–24].

Populations in the Americas are the result of admixture between Native Americans, West Eurasians and Africans; however, we are just beginning to understand the finer details of this process and its genetic correlates [25–27]. Thanks to large consortia, new studies analyzing full genomes are being published [28]. It is of biomedical and anthropological interest to survey admixture in these populations, which would help to elucidate the unique Latin American genetic landscape.

Early evidence of people in South America is seen at sites such as Monte Verde in Chile [29], with people arriving before 12,000 years ago (ya) [30], and a rapid settlement of the continent is supported by DNA studies [31](Bodner et al. 2012), mostly explained by a coastal route [29, 31–34].

Argentina is a country of a large spectrum of climates and geographical features: from subtropical rainforests in the North East to the glaciers in the Andes and the South, and from the highest peak outside Asia (the Aconcagua) to the flats of Chaco and Pampas. Evidence that hunter-gatherer groups were well settled in the North West and Center-West regions by 10,000 ya is shown by the presence of older sites such as Inca Cueva 4 (10,620 ya), Pintoscayoc (10,200 ya), Gruta del Indio (10,3500) and Agua de la Cueva (10,950) [35], and archaeological industries such as La Fortuna (8500 ya), Intihuasi (8000 ya) and Quebrada Seca 3 (9.790 ya) [36, 37]. The first signs of domestication in the North West date to near 4000 ya, seen in the sites of Inca Cueva, Huachichocana and Puente del Diablo. By 2500 ya there was the development of villages based on herding and agriculture as well as ceramic and metallurgic technology. Early sedentary villages are spread all over the North West, and between 1400 and 1100 ya there was the Aguada style expansion (simultaneous with Tiahuanaco-Wari in Central Andes, sharing characteristics such as the hallucinogenic complex and symbolism), throughout the North West except on the Puna where there was the interaction circuit Yavi-Isla that involved the North of Chile and the South West of Bolivia. In 1476 the Tawantisuyo expanded along the Andes as far South as to the present day Mendoza province in the Center-West.

The North West of Argentina (NW) was one of the most densely populated areas of the country in pre-Columbian times [38]. Its contact with Europeans dates back to the founding of the first European city in Argentinian territory, Santiago del Estero, in 1533 [39]. The Chaco area is a vast plain of which there is little archaeological data of its first settlers except that it has been inhabited for about 4,000 or 5,000 years, before which it was a swamp [40]. Native Americans from the Argentinian Chaco successfully repelled colonization, including the pre-Hispanic Tawantisuyo, until the military expeditions sent between the years 1884 and 1911 [41], with the exploitation of its forests for exotic wood the initial economic attractant for the region [42] and, in the 1920s, oil exploitation in its West. The Center-West in colonial times was part of the Capitanía General de Chile and was an important part of the route that brought goods from the port of Buenos Aires into Chile and then Peru. Until the creation of the Virreinato del Río de La Plata in 1776, the colonial control nuclei were Santiago de Chile (for the Center-West) and Lima (for the rest of present-day Argentina). Founded in 1580 and currently the capital of Argentina, Buenos Aires was an entry port for people and goods, smuggled at first and later legally transported once it became the capital of the Virreinato. Slave ships introduced Africans until 1812 [43] and there was massive immigration from Europe from the 1870s until the 1950s [44]. This was a large transoceanic migration considering the relative size of the source and receiving populations [45, 46]. As mentioned by Motti et al. [47], in the 1914 national census, 30% of Argentina's inhabitants had been born abroad but were distributed unevenly across the country: 50% of the people in the city of Buenos Aires, 30% in the provinces of Buenos Aires and Mendoza and only 2% in the provinces of Catamarca and La Rioja (North West region) were foreign-born.

Historical and genetic evidence demonstrate that the geographical distribution of the massive European wave of immigrants to Argentina was concentrated in the Pampas region of the Center-East, plus the North-East of Argentina. These patterns have been studied through uniparental lineages, both in the Creole population and the Native American communities [48–59] and in the differing ancestry proportion contribution from the Americas, Europe and Africa [23, 60–62], but their resolution was only of a continental level. The aim of this paper is to describe Argentina's genetic composition and population structure, taking into account its geographical and historical diversity. We are especially interested in possible regional differences in the Native American component of these populations, as well as those in both the sources and extent of foreign migration.

## Material and methods

### Sampling and generation of genotype data

**Fig 1:**
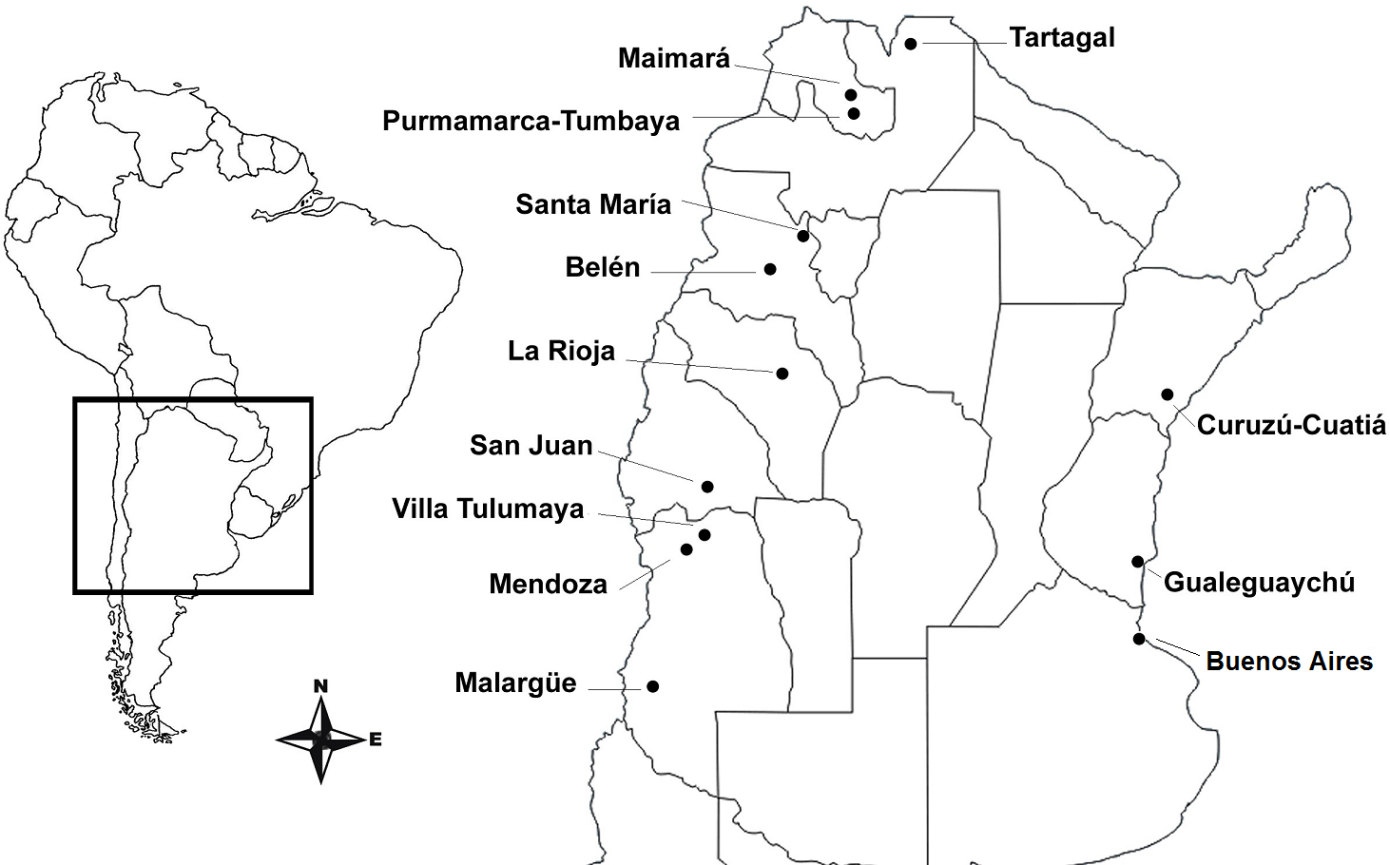
Distribution of the sampling locations in Argentina. Dots show the sampling locations included in this study.

We collected blood samples from over 3,300 participants from 17 Argentinian cities and small towns over multiple sampling efforts between 2006 and 2013. Genomic DNA was extracted from whole blood and de-identified for subsequent genotyping, sequencing and analysis. Written informed consent in Spanish was obtained from all participants under ethics approvals and permits granted by the Comité de Ética del IMBICE, and included local governmental approval from each of the 11 provinces included in the study. Donors answered a short genealogical survey to identify recent migrants from other locations or countries. Given the historical and cultural differences between regions of Argentina, these locations were grouped as shown in S1 Table. We genotyped a subset of 391 samples from 12 populations on Illumina Human Exome Beadchip v1.0 (HumanExome-12v1_A) at the Hussman Institute for Human Genomics, University of Miami. Sampling locations and summary data for the populations included in the study are detailed in S1 Table and shown on Fig 1. Using PLINK v1.07 software, we applied the following filters to the genotype data for all downstream analysis. We removed individuals with a missingness rate of > 0.1 (excluding n = 2 individuals), and SNPs with a call rate < 0.9 and missingness > 0.1. We excluded sites that were monomorphic in our sample, sites with a minor allele frequency (MAF) < 0.01, removed SNPs in high linkage disequilibrium (removing one of a pair of SNPs with r2 >0.8). Finally, we randomly removed one of a pair of individuals (excluding n = 8 individuals) demonstrating direct or distant relatedness, as determined as having pairwise pi_hat > 0.5, given that algorithms for estimating identity by descent in non-homogenous populations give a systematically inflated degree of relatedness among individuals of the same group [63]. After applying our filters, we were left with 29,145 of the 247,870 SNPs segregating in 381 individuals.

We used public data from the 1000 Genomes Project as reference populations [64], specifically the Luhya in Webuye, Kenya (LWK), Yoruba in Nigeria (YRI), Afro-Caribbean (ACB), African Americans from the US South West (ASW), Finnish (FIN), British (GBR), individuals of European Ancestry from the South West US (CEU), Tuscans (TSI), Iberians (IBS), Puerto Rican (PUR), Colombians from Medellín (CLM), Mexicans from Los Ángeles (MXL), Peruvians from Lima (PEL). Since these panels are trio data, we excluded the children, applied the same QC filters of our dataset and looked up strand flips before merging. After merging, there were 21,847 SNPs.

### Principal component and population diversity by distance analysis

The SNPRelate R package [65] was used to perform principal components analysis (PCA). Color schemes for plots were chosen from RColorBrewer. Pairwise Fst were calculated with the Weir and Cockerham formula [66] with the SNPRelate package [65]. Correlation between pairwise Fst and linear population distances was assessed with Pearson's correlation coefficient.

### Structure analysis

We used block relaxation algorithm as implemented in ADMIXTURE [67] to estimate ancestry proportions for each individual given K ancestral populations, where K was incremented from 3 to 10. We used the default setting (folds = 5) to perform ADMIXTURE’s cross-validation procedure for evaluating fit of different values of K (S1 Fig), where K = 9 showed the lowest error estimates (CV = 0.51).

### Integration of ancestry proportions and geographic coordinates

We used Surfer® software to geographically visualize the spatial distribution of average proportions of ancestry and we estimated the correlation between distance to Buenos Aires and percentage of Native American ancestry with Pearson's correlation coefficient.

From the clustering patterns observed in the ADMIXTURE analysis, a clear pattern could be distinguished between clusters and the location of sampling of different groups.

### TreeMix analysis

We used TreeMix v1.12 to infer patterns of population divergence and migrations [68]. First we ran it with all our samples, pooled by region and chose the CEU and GBR as the root position using the -root flag. We accounted for linkage disequilibrium with the -k flag using block of 20 SNPs and performed a round of the hill-climbing to optimize migration edges with the -climb flag. We included information about known migration events using the -cor_mig flag [69]. The percentage was estimated from the Admixture results and the putative sources were selected from historical information. Those events were defined as a 0.5 flow from TSI to East, 0.4 from IBS to the Center-West, and 0.2 IBS to the North East and to the North West.

## Results and discussion

**Fig 2:**
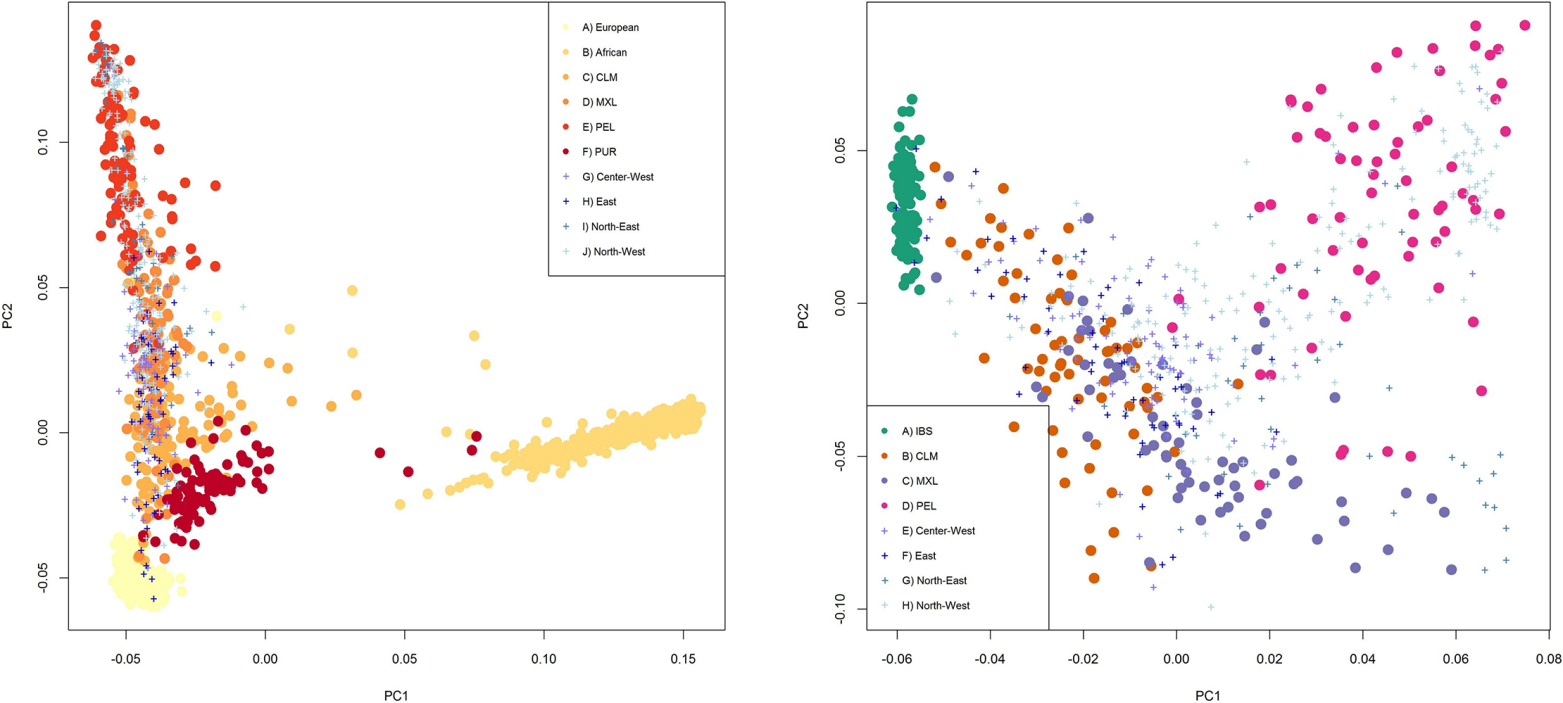
Principal component analysis. On the x axis is PC 1 while PC2 is the y axis. Plus symbols represent Argentinian samples and circles are for reference panels. Fig 2a (left) Argentinians with YRI and LWK for African references (“African”), IBS and TSI for European references (“European”) and the PEL, MXL, PUR and CLM as a Latin American references. Fig 2b (right) samples from Argentina with IBS, MXL, CLM and PEL.

On Fig 2 we represent the Principal Component Analysis (PCA) results (Fig 2). First, we used the YRI and LWK for African references, the IBS and TSI for European references and the PEL as a Latin American reference that has samples with high Native American ancestry (Fig 2A). The x axis, PC1, separates African samples from the rest while the y axis, PC2 shows Europe and the samples with highest Native American ancestry in each extreme, with the remaining admixed samples distributed along. Then, we removed the African, the TSI and all admixed individuals that had over 3% African ancestry and added the MXL and CLM panels that fit this criterion, so that we could have a better understanding of differences among the Native American component of these populations (Fig 2B). PC1 (x-axis) shows us the spectrum of admixture between full European ancestry near -0.6 while near 0.8 we have the Native American extreme. PC2 (y axis) shows that the samples with a high Native American ancestry from the North West segregate from MXL and overlap with PEL while those from the North East do not group with PEL and overlap with the MXL. Argentinians and Colombians overlapping with Mexicans is due to the low resolution of our SNP set; CLM and MXL segregate from each other in PC space when denser sets are assayed, as shown in by Gravel et al. [28].

As expected, there was a significant correlation between linear distance and Fsts (r = 0.46, p < 0.001). S2 Fig shows this correlation and S2 Table shows the pairwise Fsts and distances between populations.

**Fig 3:**
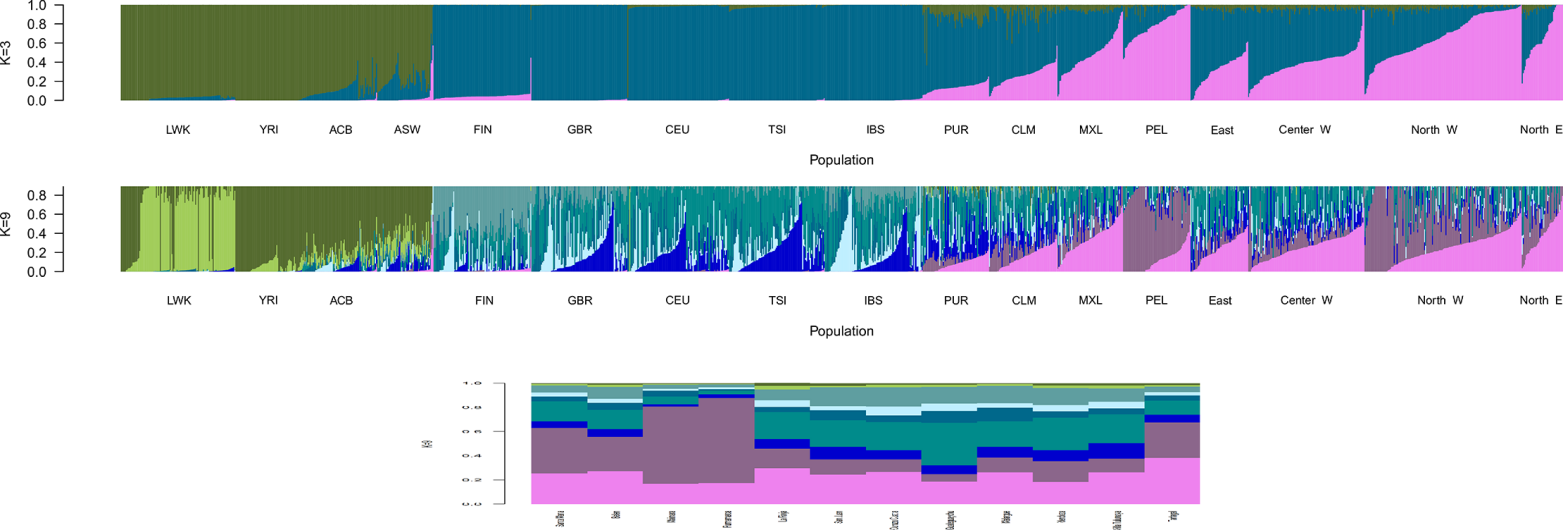
Admixture results. With 3 (K = 3) (Fig 3a, top), 9 groups (K = 9) (Fig 3b, middle) and ancestry components averaged across individuals in each per population (Fig 3c, bottom). In K = 9 there is an Andean component among the Peruvians (PEL) and the Argentinians from the North West (ANW).

Through Admixture analysis [67] we can see the admixture at both a populational and individual level (Fig 3). In Argentina, Native American ancestry is highly anti-correlated to distance to Buenos Aires (r = 0.99; p < 0.0001), where the highest percentages are the farthest from the capital. The spatial distribution of this component is shown on S3A and S3B Fig. We show the spatial distribution of each of the remaining components on S3C to S3I Fig. As mentioned in the Introduction, Buenos Aires was the entry port for the large immigration that arrived between the last decades of the 19th century and a few years after the World War II, many of whom were rural workers and merchants, who settled in the Capital and areas that were suitable for agriculture and stock breeding, mainly the Pampas and the East. This has been previously reported by surname studies [70].

The highest African percentages are on the Center West of the country, mainly Mendoza and the southernmost populations of the North West, San Juan and La Rioja. These locations were along the route that took slaves from Buenos Aires into Chile and Peru, and they had a much lower density of Native population at the time of the Conquistadors, so it was expected that they would be the ones with the highest African ancestry. This component is represented by both West African (represented by Nigerian Yorubas) and Bantu (represented by the Kenyan Luhyas) ancestries, the second slightly larger than the first. The presence of a Bantu component is explained by the slave routes that reached the La Plata basin, about half of them directly from Africa -mostly ships from Mozambique, Loango and Angola, and some from Bight of Biafra- and the other half from Brazil, where the majority came from Luanda and Benguela with a minority from Bight of Benin [43]. Also, matches to Mozambican mitDNA sequences have been found in Brazil and Santo Domingo [71–73], in concordance to our Bantu findings since these areas were affected by the Bantu expansion [74–76].

The highest Native American percentages are on the Chaco and the extreme North West. This concurs with what is known from the archaeological records -the area with the highest populational density in prehispanic times was the North West- and the fact that the Chaco was the last territory to be effectively colonized by the Argentinian government, with few Argentinean migrants to it after the oil industry established wells in the mid-1920s [77]. Hunter-gatherer Native American groups live in the Chaco and many of them even today do not speak Spanish [78].

In concordance with previous studies [79] we see different European components, with mainly a North-European and Southern-European distinction. In the Argentinian data we find a mostly a Southern-European component, which is concordant with the main immigration sources.

The North West, is part of the broad Central Andes cultural area in South America. It was part of the Kollasuyo during the Tawantisuyo and took part in other pre-hispanic cultural phenomena such as Tiawanako-Wari, the archaeological complex Yavi-Isla and Aguada 1400 ya [80], it is interesting to point out that the Native American component in these samples is Andean, different from the one found in the Chaco and other areas of the country. The Inka were not able to conquer the Chaco, they were repelled by the Guarani, Wichi, Chorote, Qom and other groups that managed to keep their independence until the 20th century [78].

**Fig 4:**
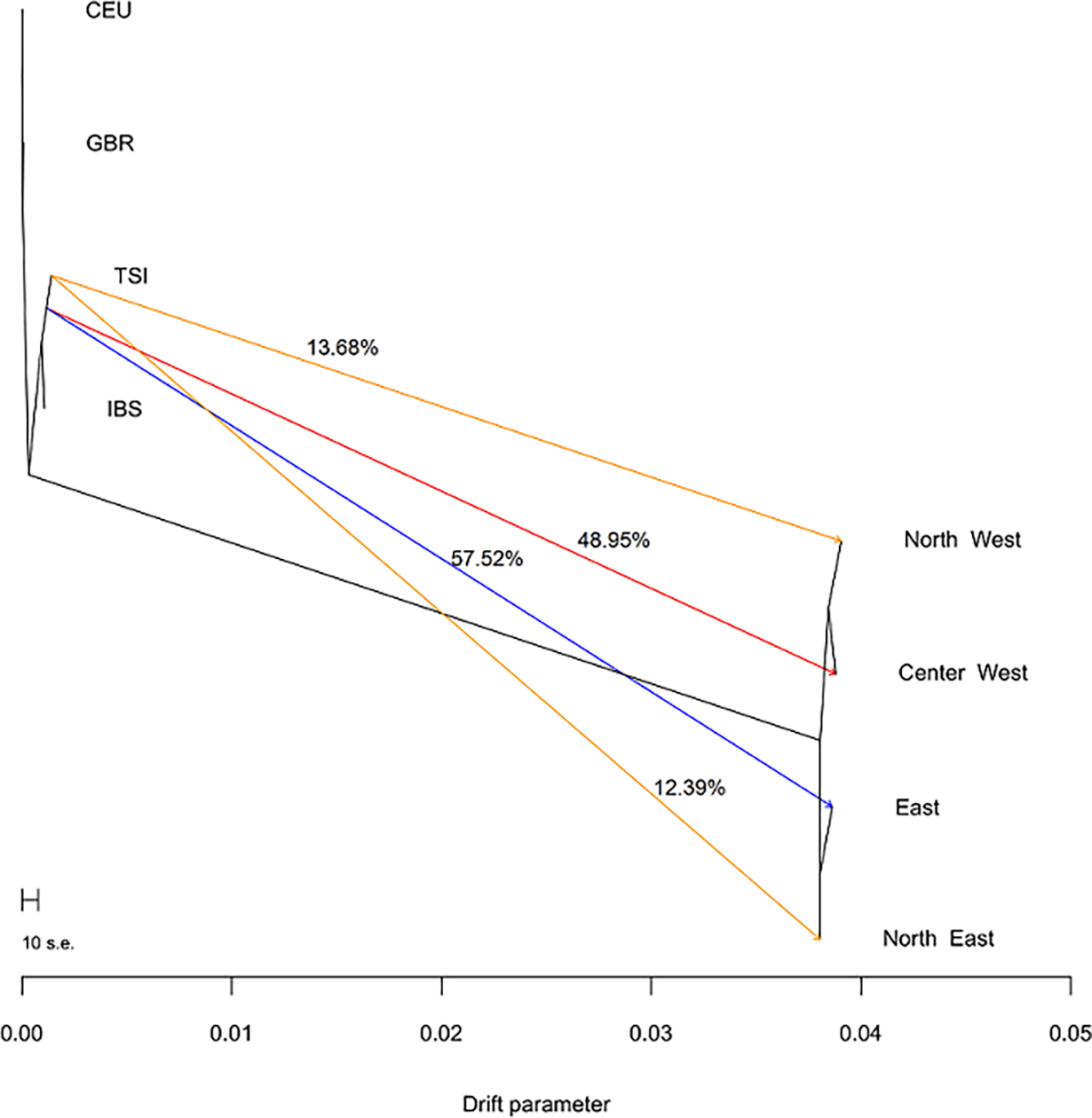
Treemix results. Fig 4 (left) Argentinian populations pooled by region and the GBR, CEU, TSI and IBS reference panels from the 1000 Genomes Project. The arrows reflect gene flow from the European source towards each Argentinian group with its percentage written.

On Fig 4 we show Treemix [68] results for the Exome Array set. We analyzed all samples sorted by region, with some European populations of the 1000 Genomes Project (Fig 4). Matching with our Admixture results, we can see how the largest European gene flow starts from the Spanish and Italian references, being the lowest for the Northern Argentinean populations, while on the East and Center-West it is high, and also coming from slightly different origins: Italian for the East and Center-West and with a Spanish influence for the North East and North West.Residual plot is available as S4 Fig.

These findings are similar to what Avena et al. [62] found, specifically regarding regional differences in ancestry components, and Campana et al. [81] found in the Center-West region and Cordoba (a province not sampled on this study). Our work allows us to have a finer resolution so that we can differentiate between different Native American ancestries, which has not been previously reported for Argentina with autosomal markers, and an African component that points to a Bantu origin instead of Western African, as seems to be the case for African ancestry in North America, the Caribbean, Colombia and Peru but not Brazil [82,83]. It is interesting to note that Brazil seems very similar regarding the sources of all three major components (except perhaps the Andean sub-component within the Native American component), with a clear difference between the proportions of African and Native American ancestries. In Brazil, the African component is much higher than in Argentina (50.8–14.7% for Brazil, 1.21–5.46% in Argentina), whereas the Native American component is much higher in Argentina (6.4–8% for Brazil, 24.84–87.49% for Argentina), which shows that even though both countries have similar migration histories in the late 19th and during the 20th centuries, the impact of the African slave trade and the interactions with the local Native Americans was much different.

As expected when considering the population history, ancestry in Argentina varies highly depending on which area is sampled, from high percentages of European ancestry to almost exclusively Native American. These results will enable future region-specific medical studies that can take advantage of Argentina’s highly diverse populations, understanding the differences within each continental source regarding each region will allow better research designs that capture this complex matrix.

## Supporting information

S1 Table Sampled populations. List of populations included with their coordinates in decimal degrees format, distance to Buenos Aires, region assigned and N.
(XLS)

S2 Table Paired distances and Fsts. (XLS)

S1 Fig CV values for K 2 to 10. The lowest CV is for K = 9.
(TIF)

S2 Fig Scatterplot between paired Fsts (y axis) and paired distance between populations (x axis). There is a correlation between paired linear distances and paired Fsts (p < 0.001).
(TIF)

S3 Fig Geographic distribution of ancestry components. a: Native American (whole) b: Andean c: Non-Andean, d: LWK, e: YRI, f: European 1, g: European 2, h: European 3, i: European 4 j: European 5
(TIF)

S4 Fig Residual plot for the Treemix results. S3 Fig shows Argentinian populations pooled by region and the GBR, CEU, TSI and IBS reference panels from the 1000 Genomes Project.
(TIF)
