# Supplementary figures and images for: Population structure in Argentina
Source: PLoS One. 2018 May 1;13(5):e0196325. doi: 10.1371/journal.pone.0196325 (PMC5929549; doi:10.1371/journal.pone.0196325)

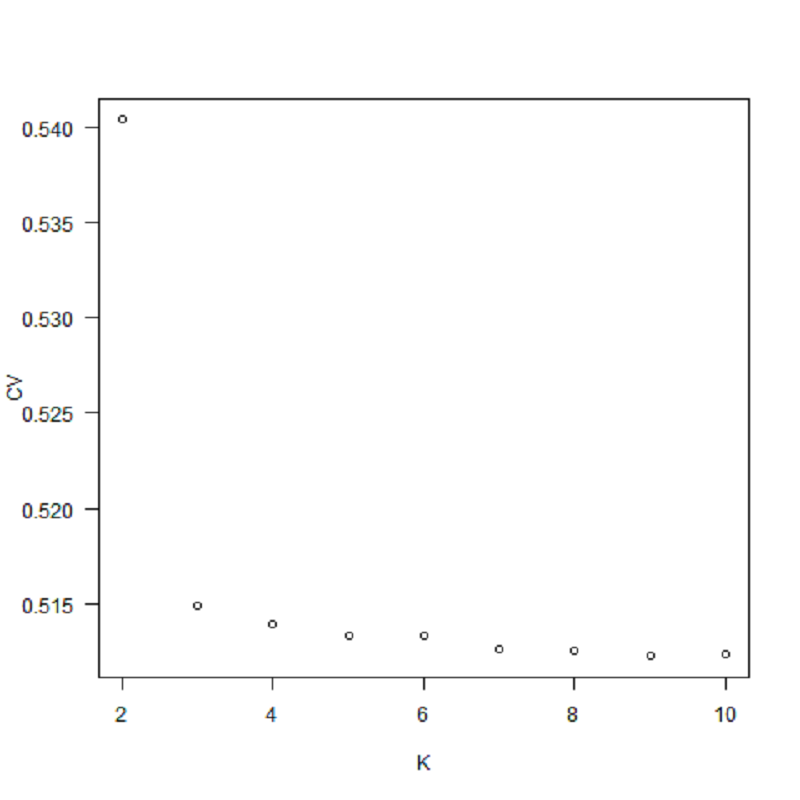

Supplement: S1 Fig — The lowest CV is for K = 9. (TIF) [file pone.0196325.s003.tif]

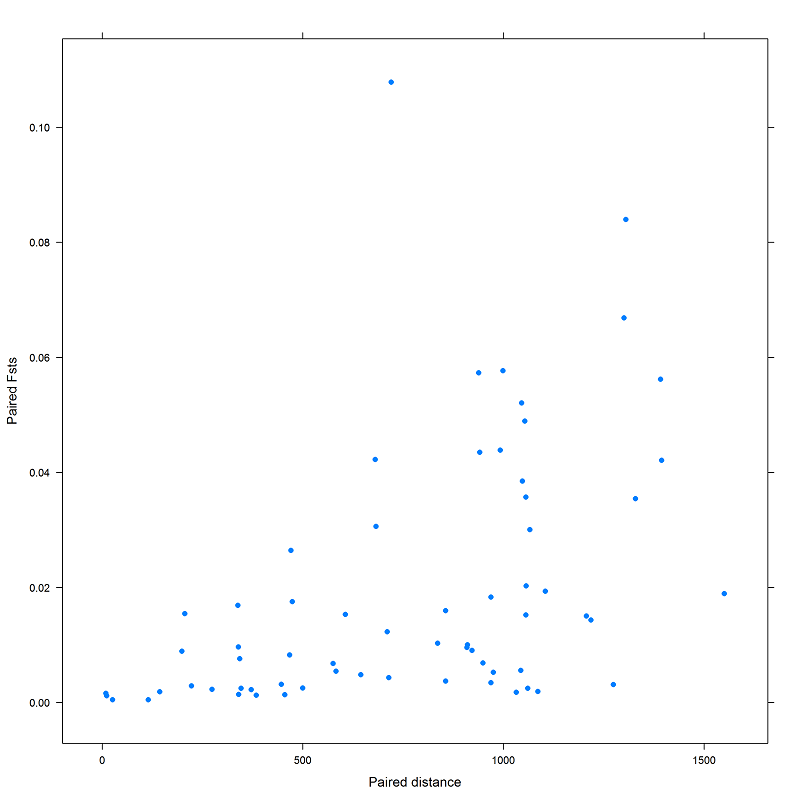

Supplement: S2 Fig — There is a correlation between paired linear distances and paired Fsts (p < 0.001). (TIF) [file pone.0196325.s004.tif]

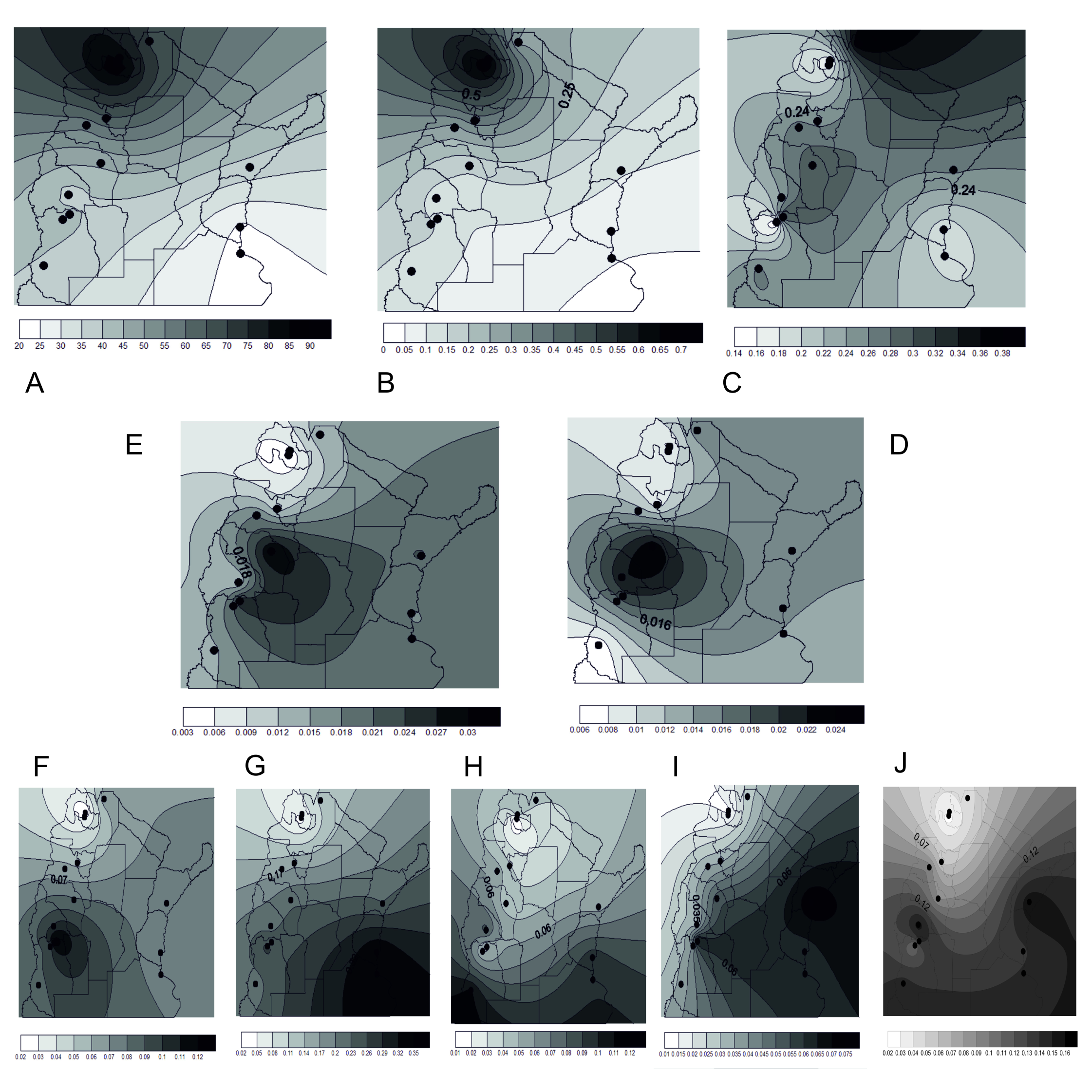

Supplement: S3 Fig — a: Native American (whole) b: Andean c: Non-Andean, d: LWK, e: YRI, f: European 1, g: European 2, h: European 3, i: European 4 j: European 5 (TIF) [file pone.0196325.s005.tif]

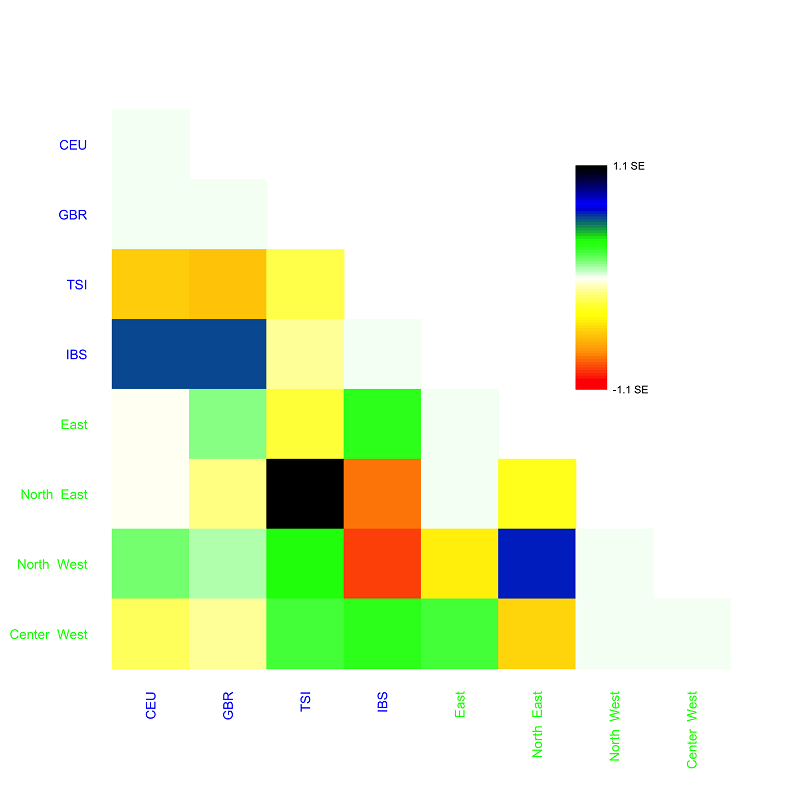

Supplement: S4 Fig — S3 Fig shows Argentinian populations pooled by region and the GBR, CEU, TSI and IBS reference panels from the 1000 Genomes Project. (TIF) [file pone.0196325.s006.tif]
